# Personalization Strategies for Increasing Engagement With Digital Mental Health Resources: Sequential Multiple Assignment Randomized Trial

**DOI:** 10.2196/73188

**Published:** 2025-11-04

**Authors:** Julien Rouvere, Isabell R Griffith Fillipo, Meghan Romanelli, Ashish Sharma, Brittany A Mosser, Theresa Nguyen, Kevin Rushton, John Marion, Tim Althoff, Michael D Pullmann

**Affiliations:** 1 Department of Psychiatry and Behavioral Sciences, University of Washington School of Medicine, 1959 NE Pacific Street, Box 356560, Seattle, WA, 98195-6560, United States, 1 206-221-5498; 2 School of Social Work, University of Washington, Seattle, WA, 98105, United States; 3 Paul G. Allen School of Computer Science and Engineering, University of Washington, Seattle, WA, 98195, United States; 4 Mental Health America, Alexandria, VA, 22314, United States

**Keywords:** sequential multiple assignment randomized trial, SMART, Mental Health America, MHA, tailoring strategies, personalization, mental health website

## Abstract

**Background:**

Although web-based mental health resources have the potential to assist millions, particularly those who face barriers to treatment, most mental health website visitors disengage before accessing resources that can help improve their mental health.

**Objective:**

We used a sequential multiple assignment randomized trial to test whether personalized tailoring improved engagement on a self-guided mental health website.

**Methods:**

Data were collected via voluntary response sampling on the Mental Health America website. Inclusion criteria included residing in the United States and viewing a postscreening survey after completing the Patient Health Questionnaire-9 (PHQ-9). Participants were randomized to 1 of 2 postscreening survey conditions: the demographics survey or the Next Steps survey, which included additional tailoring questions assessing perceived need and participants’ intended next steps. Participants who viewed the following screening results page were subsequently randomized to 1 of 5 conditions that displayed nontailored or tailored messages and featured resources, as well as persistent general resources that did not vary by condition. Data were analyzed using logistic regressions predicting disengagement and clicks on featured resources (versus persistent general resources) by condition.

**Results:**

Adding questions to inform tailoring significantly increased the odds of disengaging by 14% (demographics survey: 25%; Next Steps survey: 27.5%; odds ratio [OR] 1.14, 95% CI 1.11‐1.16; *P*<.001). Among participants who viewed a postscreening survey (n=169,647), 87,712 participants were randomized to the demographics survey condition, and 81,935 participants were randomized to the Next Steps survey condition. Among participants who submitted the demographics survey (n=38,490), tailoring resources to demographics reduced the odds of disengaging by 10% (OR 0.90, 95% CI 0.87‐0.94; *P*<.001) and, among those who engaged, increased the odds of clicking a featured resource versus a persistent general resource by 90% (OR 1.90, 95% CI 1.79‐2.01; *P*<.001). Among participants who submitted the Next Steps survey (n=34,204), tailoring messages to perceived need (*P*=.33), tailoring resources to intended next steps (*P*=.51), and a combination of both (*P*=.52) did not significantly reduce the odds of disengaging compared with the nontailored condition. However, tailoring resources to intended next steps and combining a tailored message to perceived need with tailored resources to intended next steps increased the odds of clicking a featured resource by 25% (OR 1.25, 95% CI 1.14‐1.37; *P*<.001) and 34% (OR 1.34, 95% CI 1.23‐1.47; *P*<.001), respectively. Tailoring resources to demographics was significantly more effective in improving engagement than tailoring to perceived need or intended next steps (*P*≤.004).

**Conclusions:**

There was a small but statistically significant cost to engagement from adding tailoring questions assessing perceived need and intended next steps. Among the strategies tested in this study, tailoring resources to demographics was the most effective strategy for increasing engagement among visitors who viewed their screening results. This study demonstrates how personalization may increase engagement with mental health websites and provides design implications for future research.

## Introduction

In 2023, 53.9% of the 58.7 million United States adults living with a mental illness received mental health treatment in the past year [1]. Self-guided mental health websites may be especially helpful for those who face structural barriers (eg, time, geographic constraints, and cost) and individual barriers (eg, stigma, low perceived need, and lack of readiness) to treatment [1-3]. Mental health websites bypass many of these obstacles by maintaining user privacy and offering accessible and free resources; they may also be preferred by some over face-to-face care [4]. For instance, the Mental Health America (MHA) [5] website annually attracts approximately 10 million visitors and provides free screening tools and resources, such as psychoeducational articles, therapist directories, and self-help tools. These resources are designed to support individuals at any stage of their mental health journey, ranging from those exploring mental health topics for the first time to those actively seeking treatment.

Helping individuals find and use quality web-based mental health resources has positive implications for future treatment decisions and stabilization [6,7]. However, visitors to mental health websites, including MHA’s, typically engage for only seconds to minutes [8]. One qualitative study of young adults who completed screening tools on MHA found that most participants did not have plans for immediate action after completing a screening tool [9]. Given the underutilization of web-based mental health resources and their potential benefits, there is a critical need to identify strategies that encourage visitors to access resources that can help improve their mental health.

Personalization may be an effective strategy for promoting engagement with mental health websites [10,11]. Research shows that tailored information sometimes influences health behaviors more effectively than nontailored information [12,13]. For example, one meta-analysis found that tailoring was associated with greater efficacy of web-based interventions in health-promoting behavior change compared with control [14]. However, whether personalization improves engagement with a real-world self-guided mental health website remains unclear.

Tailoring in this study was based on factors associated with engagement identified by previous research. In preparation for this study, our team conducted unpublished micro-randomized trials (MRTs) testing design elements that might optimize engagement from April 2022 to September 2023. These MRTs and previous research indicated different usage patterns by age group (eg, older adults tend to access resources related to treatment, younger adults are more likely to use mental health websites than older adults) [6]. In addition, our MRTs indicated that lesbian, gay, bisexual, transgender, and queer (LGBTQ+) individuals tended to have lower engagement rates than visitors who did not identify as LGBTQ+, despite evidence that mental health websites are vital sources of information for LGBTQ+ individuals [15]. One literature review found that tailoring content to LGBTQ+ identity was important for LGBTQ+ people who used digital mental health interventions [16].

Tailoring was also informed by the Health Action Process Approach (HAPA), a stage-based theoretical framework that proposes that health behaviors are adopted and maintained through a process of developing behavioral intentions (ie, the motivation phase) followed by planning to action (ie, the volition phase) [17,18]. Mechanisms that influence an individual’s progression through these stages include, for example, outcome expectancies, perceived risks, and perceived need [17-19]. Research indicates that perceived need may be associated with the formation of behavioral intentions to engage with eHealth interventions [19] and mental health websites [20]. The aim of tailoring to perceived need in the current study was to facilitate engagement by encouraging individuals with low perceived need to transition from preintention to intention and to provide positive reinforcement to those with a perceived need to take action to improve their mental health. In addition, the purpose of tailoring to visitors’ intended next steps on the website was to provide resources relevant to each individual’s stage of change. For instance, individuals who wanted to learn more about depression were provided with links to psychoeducational articles, and those who wanted to find a treatment provider received links to therapist directories and articles related to seeking treatment.

The goal of this study was to determine whether engagement with a mental health website could be improved by tailoring content to age, LGBTQ+ status, perceived need, and intended next steps via a sequential multiple assignment randomized trial (SMART) [21]. Before this study, visitors who completed the Patient Health Questionnaire-9 (PHQ-9) [22] were shown an optional demographics survey before the screening results page. However, acquiring information to inform tailoring necessitates the addition of tailoring questions, and previous research shows that longer surveys are associated with higher dropout [23]. For this study, we used the Next Steps survey, which included 2 tailoring questions before the items on the demographics survey, to assess whether the addition of tailoring questions hindered engagement. Submitting the demographics or Next Steps survey took visitors to the screening results page, which showed nontailored or tailored messages and featured resources (ie, prominently displayed links to resources on MHA). Additional persistent general resources were displayed underneath featured resources on the screening results page to all participants independent of condition. This study addressed the following aims:

1. Does adding tailoring questions negatively impact engagement?
2. Does personalization to demographics improve engagement?
3. Does tailoring to perceived need, intended next steps, or a combination of both improve engagement?
4. Among the strategies examined in this study, which is the optimal tailoring strategy?

These findings will inform the implementation of tailoring on the MHA website, offer novel insights on how to optimally deliver information to people seeking web-based mental health information, and provide empirical evidence on the effectiveness of tailoring on a large, real-world self-guided mental health website.

## Methods

### Overview

A session was defined as activity on the MHA website from a unique IP address bounded by 30 minutes of inactivity. Visitors with ≥30 minutes of inactivity were considered to have disengaged, and only the first session of each unique IP address was used for analyses. Data were collected from 258,383 sessions from December 1, 2023 to February 6, 2024 and from April 1, 2024 to April 30, 2024 on MHA. Data from February 7, 2024 to March 31, 2024 were excluded due to minor programming errors that were subsequently resolved by MHA.

### Participants

Participants were recruited on MHA through voluntary response sampling. Inclusion criteria were residing in the United States and viewing a postscreening survey after completing the PHQ-9 (n=169,647). See Multimedia Appendix 1 for MHA’s data cleaning procedures.

### Procedure

#### Overview

**Figure 1.**
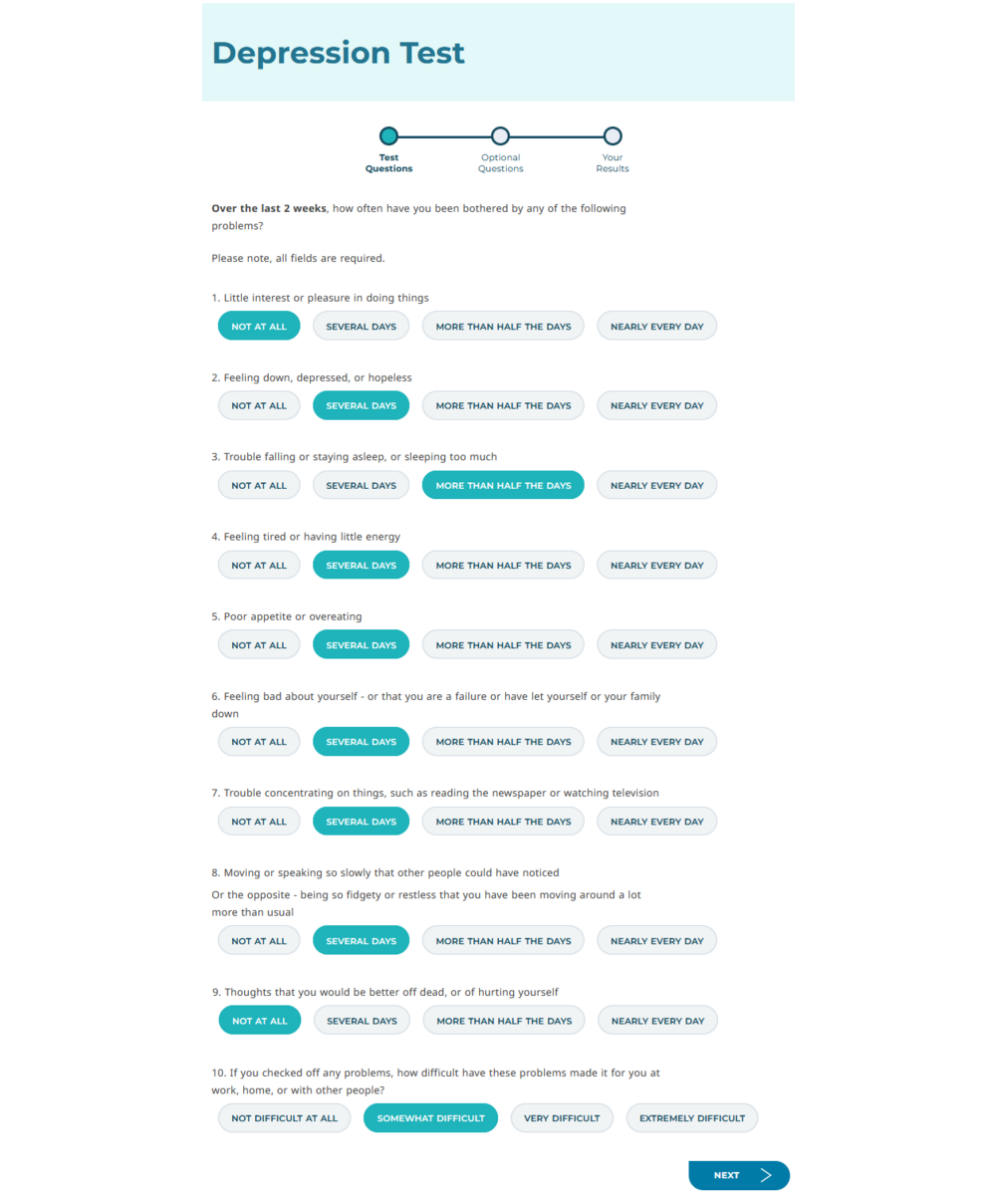
Web page 1: participants began on a web page displaying the PHQ-9, which did not vary by condition; the figure shows sample responses selected. PHQ-9: Patient Health Questionnaire-9.

**Figure 2.**
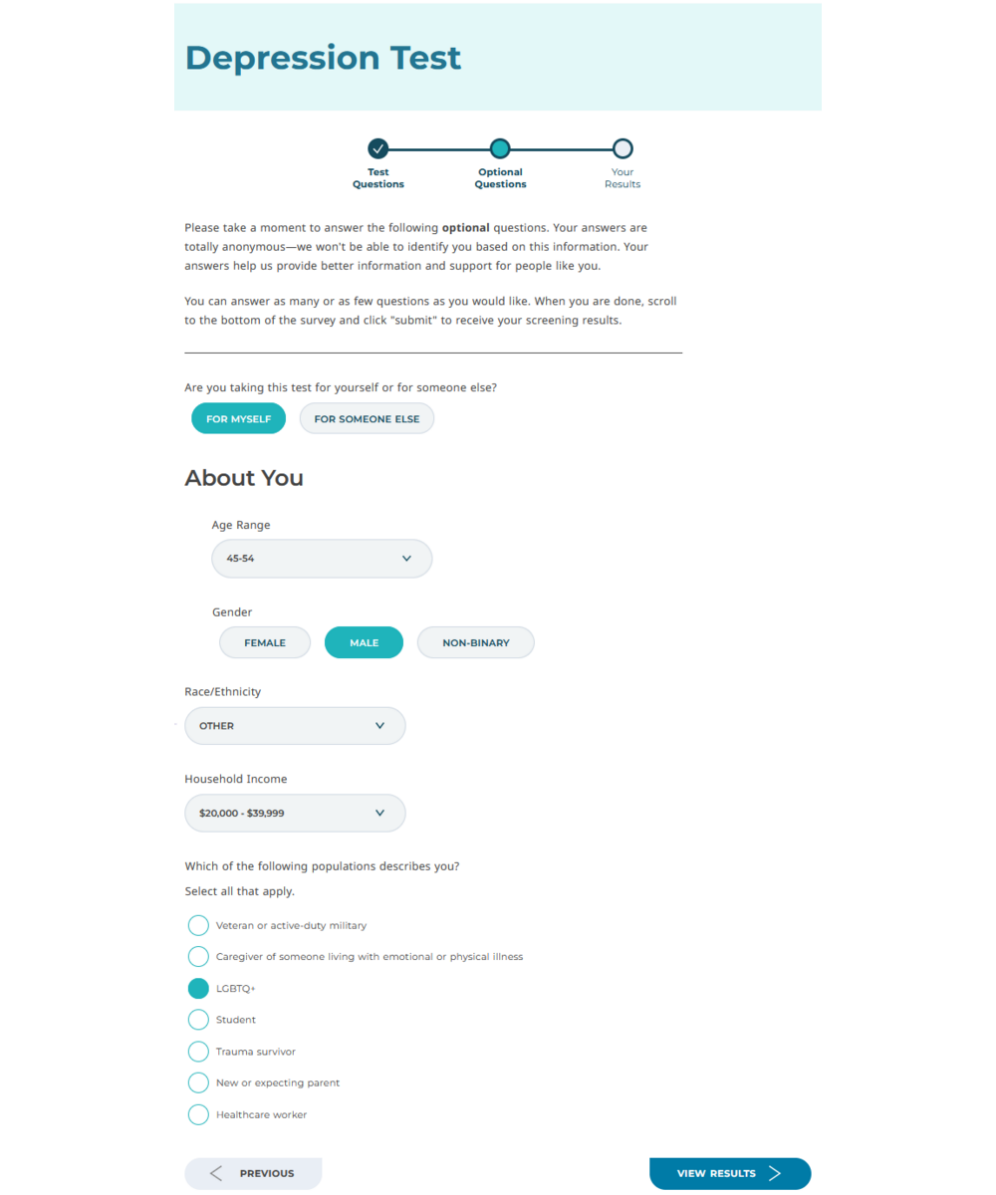
Web page 2: after submitting the PHQ-9, participants were shown a postscreening survey (ie, demographics survey or Next Steps survey); the figure displays select items from the demographics survey with sample responses selected. PHQ-9: Patient Health Questionnaire-9.

This study used a SMART design. Participants could progress through 3 web pages displaying: (1) the PHQ-9, (2) a postscreening survey, and (3) their screening results. Participants began on the PHQ-9 web page (see Figure 1), which did not vary by condition. Participants were randomized via simple randomization to 1 of 2 Level 1 conditions (ie, demographics survey or Next Steps survey) upon clicking the PHQ-9 web page, on which a link to the appropriate postscreening survey was generated. Upon completing the PHQ-9 and clicking the “Next” button, participants were taken to the postscreening survey web page, which displayed either the demographics survey or Next Steps survey (see Figure 2 for a sample demographics survey web page).

Postscreening survey items were optional. Participants who clicked the “View Results” button at the bottom of the postscreening survey progressed to the screening results page and were randomized to 1 of 5 Level 2 conditions. Participants who submitted the demographics survey were randomized to 1 of 2 possible conditions: (1) nontailored or (2) tailored resources to demographics (TR-D). Participants who submitted the Next Steps survey were randomized to 1 of 4 conditions: (1) nontailored, (2) tailored message to perceived need (TM-PN), (3) tailored resources to intended next steps (TR-INS), or (4) tailored message to perceived need + tailored resources to intended next steps (TM-PN+TR-INS).

The following descriptions of Level 2 conditions provide examples of messages and resources shown in each condition. For a complete list of messages and featured resources, see Multimedia Appendix 2.

#### Nontailored Condition

Participants from either Level 1 condition could be randomized to the nontailored condition on the screening results page. The nontailored condition included nontailored messages and nontailored featured resources. A nontailored message showed visitors a brief description of their PHQ-9 results, which was shown across all conditions. For example, participants with a PHQ-9 score from 20‐27 were shown, “Based on your responses, you may have symptoms of severe depression. This result is not a diagnosis. A doctor or therapist can help you get a diagnosis and/or treatment.” Nontailored featured resources comprised 4 featured resources (ie, links to resources on MHA that were prominently displayed in large, colorful bubbles across all conditions), shown in random order, that were selected by MHA’s algorithm as the most popular resources accessed by visitors who complete the PHQ-9.

#### Tailored Resources to Demographics (TR-D) Condition

Participants who completed the demographics survey could be randomized to the TR-D condition, which showed participants a nontailored message (ie, brief description of PHQ-9 results) and featured resources tailored to age and LGBTQ+ status. A pool of 3 to 4 possible resources for each age group was chosen by an algorithm that identified the most popular resources within each age group. Participants who provided their age and did not self-identify as LGBTQ+ were shown all possible resources for their age group. For example, a participant between 25‐45 years old who did not self-identify as LGBTQ+ was shown the following links in random order: “I can’t do anything right—depression,” “I feel numb,” “I can’t get out of bed,” and “Does depression go away on its own?”

**Figure 3.**
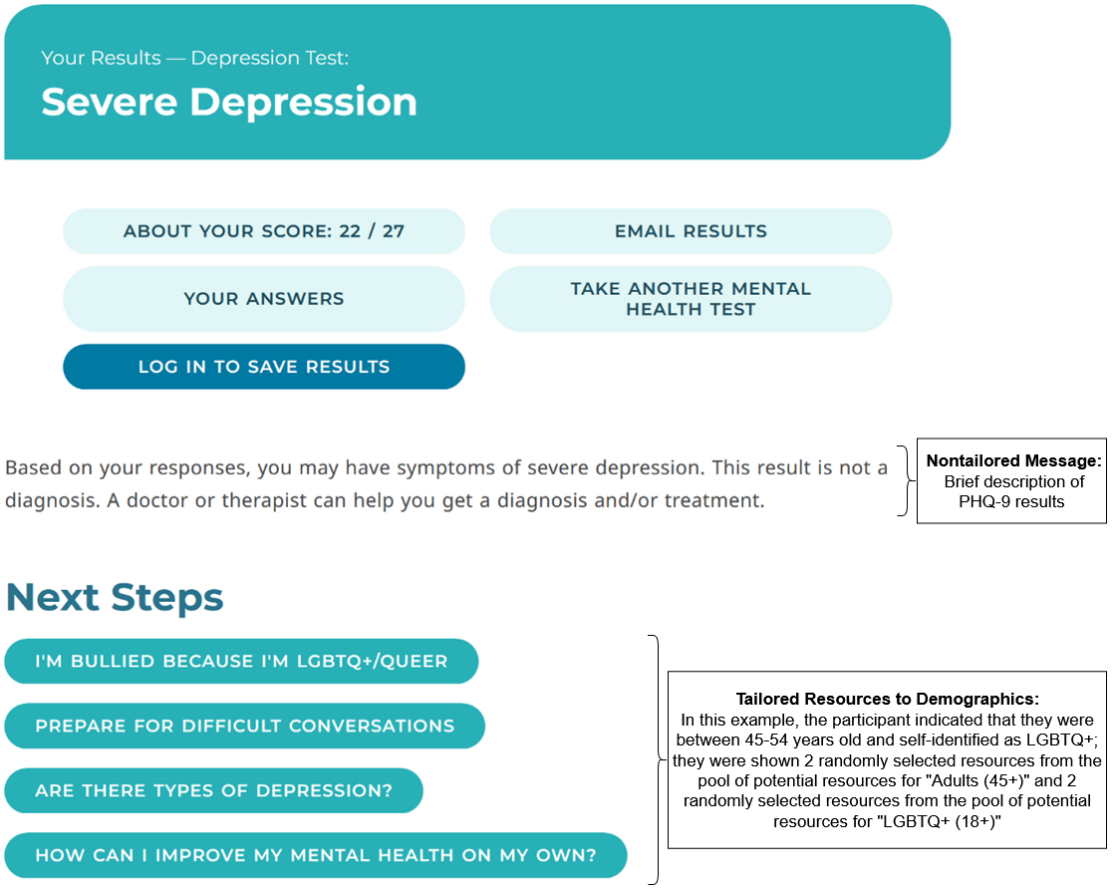
Sample screening results page: tailored resources to demographics condition, which included a nontailored message and featured resources tailored to age and LGBTQ+ status. LGBTQ+: lesbian, gay, bisexual, transgender, and queer.

Tailored resources to LGBTQ+ status comprised resources on MHA that cater to the LGBTQ+ population. Participants who self-identified as LGBTQ+ and did not provide age were shown all 4 possible LGBTQ+ resources: “I’m bullied because I’m LGBTQ+/queer,” “Q-chat space,” “Prepare for difficult conversations,” and “How do I find LGBTQ-friendly therapy?” LGBTQ+ participants who also provided age were shown 2 age-tailored resources and 2 LGBTQ+ resources; the 2 LGBTQ+ resources were selected from a pool of 4 possible resources for participants under 18 years or 3 possible resources for those 18 years and older. See Figure 3 for an example of a screening results page for a participant randomized to the TR-D condition who was between 45‐54 years old and self-identified as LGBTQ+.

#### Tailored Message to Perceived Need (TM-PN) Condition

**Figure 4.**
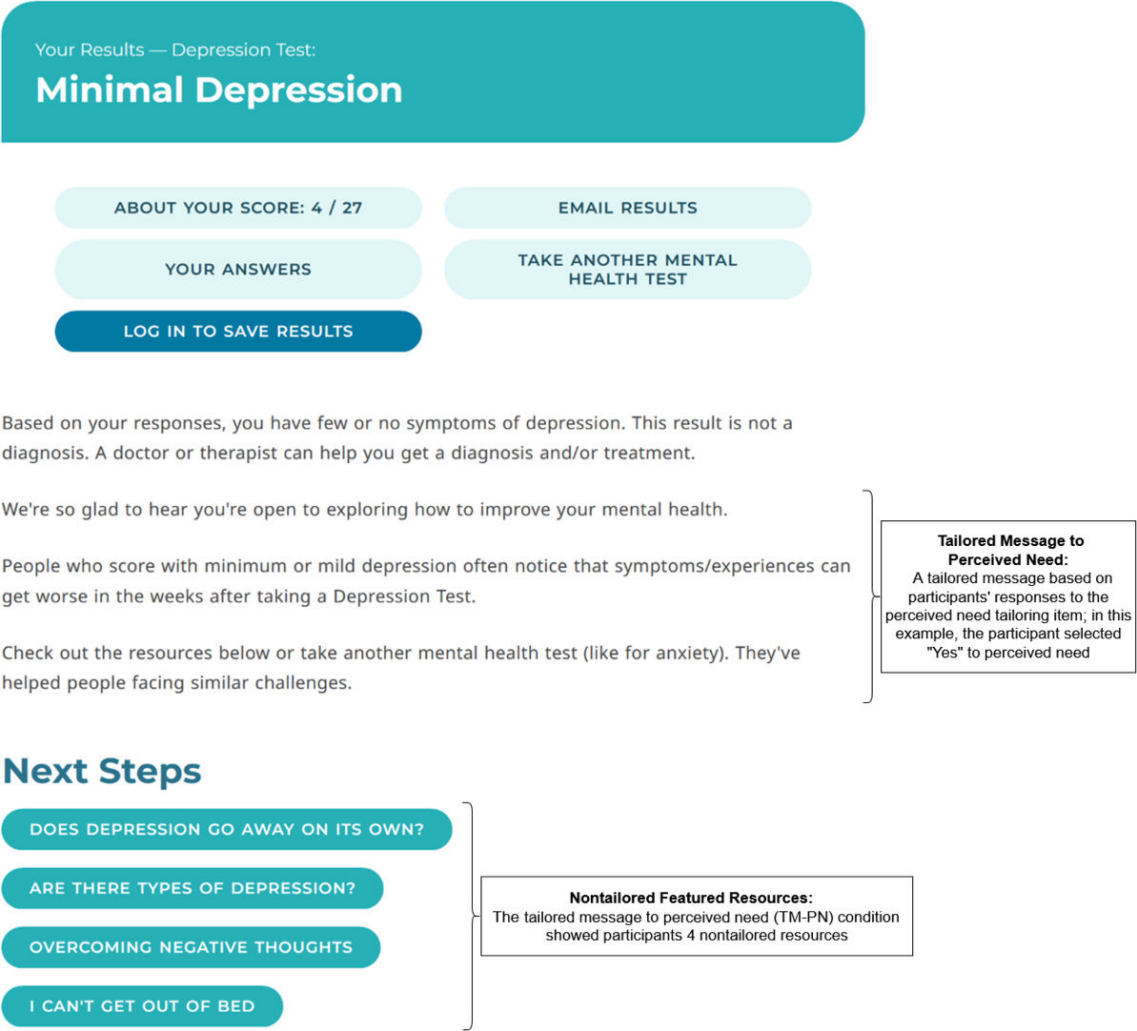
Sample screening results page: tailored message to perceived need condition, which included a tailored message to perceived need and nontailored featured resources.

The TM-PN condition was 1 of 4 conditions available to participants who completed the Next Steps survey. Tailored messages were based on participants’ responses to the perceived need item, followed by text based on their PHQ-9 results. The TM-PN condition displayed nontailored featured resources. See Figure 4 for an example of a screening results page for a participant randomized to the TM-PN condition who endorsed “Yes” to perceived need.

#### Tailored Resources to Intended Next Steps (TR-INS) Condition

Participants who completed the Next Steps survey could also be randomized to the TR-INS condition, which included a nontailored message and 4 featured resources tailored to participants’ responses to the intended next steps item. For example, a participant who selected “Understand what depression is like (reading articles)” was shown “What is depression really like?,” “Am I depressed or just sad?,” “Are there types of depression?,” and “Am I broken?” in random order.

#### Tailored Message to Perceived Need + Tailored Resources to Intended Next Steps (TM-PN+TR-INS) Condition

**Figure 5.**
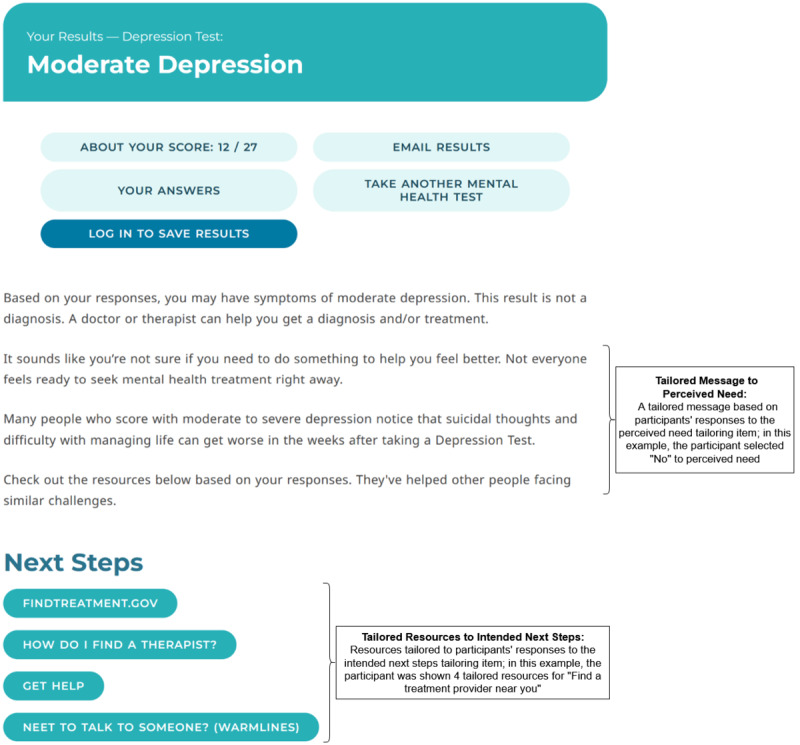
Sample screening results page: tailored message to perceived need + tailored resources to intended next steps condition.

The TM-PN+TR-INS condition was available to participants who completed the Next Steps survey. The TM-PN+TR-INS condition included a tailored message to perceived need and 4 featured resources tailored to participants’ intended next steps. See Figure 5 for an example of a screening results page for a participant randomized to the TM-PN+TR-INS condition who endorsed “No” to perceived need and “Find a treatment provider near you” as their intended next steps.

#### Persistent General Resources

**Figure 6.**
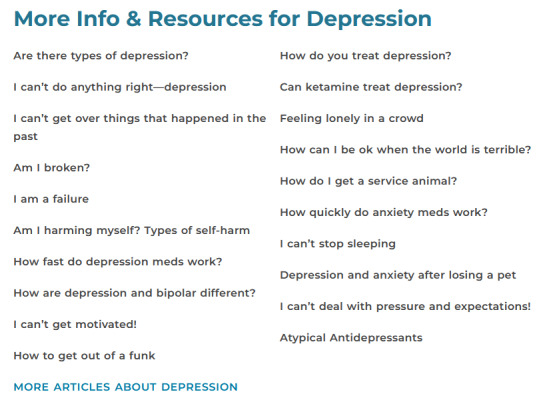
Examples of persistent general resources (shown to all participants independent of condition) displayed underneath featured resources on screening results pages.

Persistent general resources included links to resources related to depression. Persistent general resources were shown underneath featured resources on the screening results page to all participants, independent of condition (see Figure 6).

#### Programming Errors

Two minor programming and execution errors occurred. All conditions were intended to receive 4 resources, but only 3 were shown to participants in the 45+ years age group in the TR-D condition, unless they also indicated being LGBTQ+. However, this only affected a small portion of the sample. People who reported that they lived in Minnesota (2.1% of the 77% of participants who provided their state of residence) were shown a Minnesota-specific resource in place of one of the featured resources.

### Measures

#### Demographics Survey

Participants were invited to provide their age range, gender, race and ethnicity, and household income. They were also asked to select applicable descriptors among veteran or active-duty military, caregiver of someone living with emotional or physical illness, LGBTQ+, student, trauma survivor, new or expecting parent, and healthcare worker.

#### Next Steps Survey

The Next Steps survey included 2 tailoring questions shown before the demographics survey items. The first question asked about participants’ intended next steps: “What is the main thing you want to do after taking this mental health test? (Select one).” Response options included, but were not limited to, “Take another mental health test,” “Understand what depression is like (reading articles),” and “Understand and manage self-harm or suicidal thoughts.” The second tailoring item assessed perceived need (“Do you feel like you need to do something to improve your mental health?”) with options of “Yes,” “No,” and “I don’t know.”

#### Engagement

Our primary engagement outcome was disengagement, operationalized as leaving the website or having ≥30 minutes of inactivity (0=engaged, 1=disengaged). We also measured engagement on the screening results page by whether participants clicked a featured or persistent general resource (0=clicked a persistent general resource, 1=clicked a featured resource).

### Analyses

#### Impact of Adding Tailoring Questions on Engagement

Data were analyzed in R Version 4.3.3 (R Core Team) [24], and all research questions were examined via logistic regressions. We assessed whether Level 1 condition (ie, demographics survey or Next Steps survey) was a significant predictor of disengaging from the postscreening survey to examine the costs, if any, of adding tailoring questions. For the remaining analyses, we used 2-part models to first examine the odds of disengaging from the screening results page by Level 2 condition. We then examined the odds of clicking a featured versus a persistent general resource among participants who chose to engage on the screening results page. Statistical significance was not impacted after corrections for multiple comparisons using the Benjamini-Hochberg procedure with a false discovery rate of 0.05 [25]. We report odds ratios (OR) with 95% CIs and *P* values.

#### Impact of Tailoring on Engagement

Among participants who submitted the demographics survey (n=38,490), we examined whether the odds of disengaging (versus engaging) and clicking a featured resource (versus clicking a persistent general resource) on the screening results page significantly differed for participants in the TR-D condition compared with the nontailored condition. Among participants who submitted the Next Steps survey (n=34,204), we tested whether engagement significantly differed for participants in the TM-PN, TR-INS, and TM-PN+TR-INS conditions compared with the nontailored condition.

#### Identifying the Optimal Tailoring Strategy in This Study

We aimed to identify the optimal tailoring strategy among those tested in this study by comparing engagement across all possible conditions on the screening results page, with TR-D set as the reference group. Conditions included nontailored (including participants from both the demographics survey and Next Steps survey conditions), TR-D, TM-PN, TR-INS, and TM-PN+TR-INS.

### Ethical Considerations

This study was approved by the University of Washington Institutional Review Board (STUDY00010958). The Institutional Review Board and the study’s external Data Safety and Monitoring Board concluded that there were no foreseeable risks associated with participation in the study, and further application for ethical approval was not necessary. MHA visitors engaged with the website naturalistically, and the website’s terms and conditions include provisions around the use of user data for research. Thus, the Institutional Review Board waived informed consent, and participants were not compensated for their involvement in the study. Data were protected under the University of Washington Institutional Review Board and were deidentified via random identifiers before data transfers between MHA and the University of Washington in accordance with a Data Security Protocol. The deidentified data were stored on a secure encrypted institutional server with access restricted to approved research personnel only.

## Results

### Sample Characteristics

**Figure 7.**
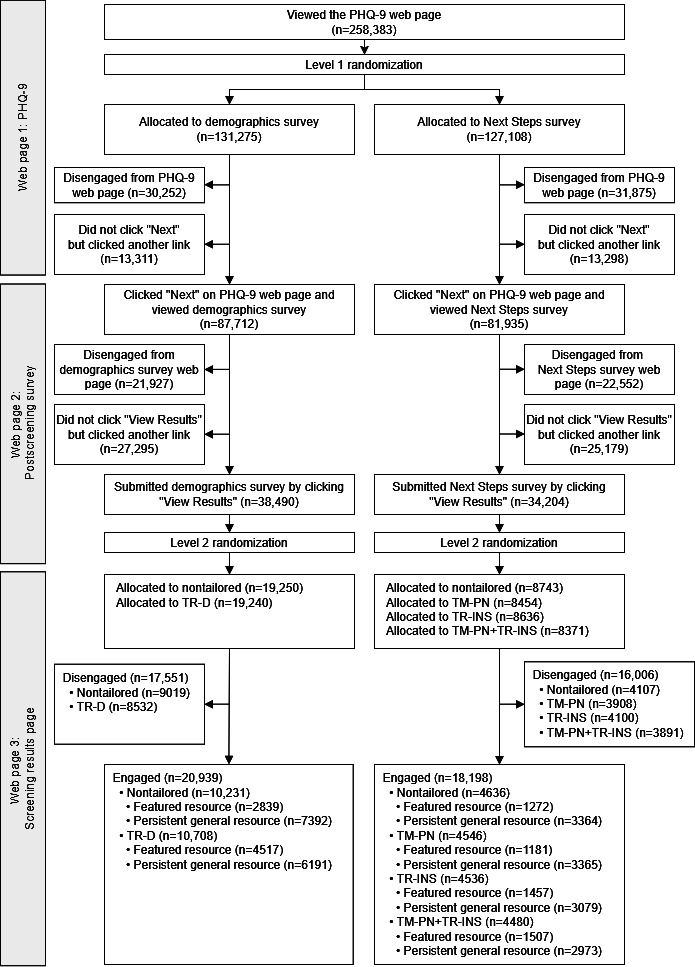
Study flow diagram. Level 1 randomization was triggered upon viewing the PHQ-9 web page; participants who completed the PHQ-9 and subsequently viewed a postscreening survey web page were included in the analytic sample (n=169,647). PHQ-9: Patient Health Questionnaire-9; TM-PN: tailored message to perceived need; TM-PN+TR-INS: tailored message to perceived need + tailored resources to intended next steps; TR-D: tailored resources to demographics; TR-INS: tailored resources to intended next steps.

Among participants who clicked the PHQ-9 (n=258,383), 131,275/258,383 (50.8%) were randomized to the demographics survey, and 127,108/258,383 (49.2%) were randomized to the Next Steps survey. See Figure 7 for the study flow diagram. Among participants who viewed a postscreening survey (n=169,647), 87,712/169,647 (51.7%) participants were allocated to the demographics survey condition, and 81,935/169,647 (48.3%) were allocated to the Next Steps survey condition. Most participants who submitted a postscreening survey (n=72,694) had moderately severe or severe depression (mean 15.9, SD 6.1). Among participants who also provided demographic data, most were White (n=37,311/69,385, 53.8%); Hispanic or Latino (n=10,218/69,385, 14.7%); Asian (n=6647/69,385, 9.6%); Black or African American (n=6532/69,385, 9.4%); women (n=43,517/71,963, 60.5%); and between 18‐24 years old (n=15,604/71,529, 21.8%; see Table 1). Among participants who responded to the intended next steps tailoring question (n=33,002), the most common responses were “I don’t want to do anything” (n=12,034, 36.5%) followed by “Take another mental health test” (n=4740, 14.4%). Among participants who responded to the perceived need item (n=33,352), 60.9% (n=20,325) of participants endorsed “Yes” to perceived need (see Table 2). Among participants randomized to a Level 2 condition (n=72,694), disengagement rates on the screening results page ranged from 44.3% to 47.5% by condition. Among those who engaged on the screening results page (n=39,137), rates of clicking a featured resource ranged from 26% to 42.2%, and rates of clicking a persistent general resource ranged from 57.8% to 74% by condition.

**Table 1.** Demographic characteristics by Level 1 condition among participants who submitted a postscreening survey.

Characteristic	Demographics survey (n=38,490)	Next Steps survey (n=34,204)	Total^a^ (n=72,694)
Race and ethnicity, n	36,827	32,558	69,385
American Indian or Alaska Native, n (%)	789 (2.1)	748 (2.3)	1537 (2.2)
Asian, n (%)	3532 (9.6)	3115 (9.6)	6647 (9.6)
Black or African American (non-Hispanic), n (%)	3402 (9.2)	3130 (9.6)	6532 (9.4)
Hispanic or Latino, n (%)	5324 (14.5)	4894 (15)	10,218 (14.7)
Middle Eastern or North African, n (%)	382 (1)	354 (1.1)	736 (1.1)
Native Hawaiian or Other Pacific Islander, n (%)	185 (0.5)	173 (0.5)	358 (0.5)
White (non-Hispanic), n (%)	20,004 (54.3)	17,307 (53.2)	37,311 (53.8)
Other, n (%)	1243 (3.4)	1089 (3.3)	2332 (3.4)
More than one of the above, n (%)	1966 (5.3)	1748 (5.4)	3714 (5.4)
Missing, n	1663	1646	3309
Gender identity^b^, n	38,151	33,812	71,963
Woman, n (%)	23,065 (60.5)	20,452 (60.5)	43,517 (60.5)
Man, n (%)	13,970 (36.6)	12,316 (36.4)	26,286 (36.5)
Nonbinary, n (%)	1116 (2.9)	1044 (3.1)	2160 (3)
Missing, n	339	392	731
Household income, n	29,265	25,879	55,144
Less than US $20,000, n (%)	5619 (19.2)	5349 (20.7)	10,968 (19.9)
US $20,000-US $39,999, n (%)	4630 (15.8)	4150 (16)	8780 (15.9)
US $40,000-US $59,999, n (%)	4282 (14.6)	3671 (14.2)	7953 (14.4)
US $60,000-US $79,999, n (%)	3624 (12.4)	3239 (12.5)	6863 (12.4)
US $80,000-US $99,999, n (%)	2916 (10)	2598 (10)	5514 (10)
US $100,000-US $149,999, n (%)	4064 (13.9)	3476 (13.4)	7540 (13.7)
US $150,000+, n (%)	4130 (14.1)	3396 (13.1)	7526 (13.6)
Missing, n	9225	8325	17,550
Age range (years), n	37,897	33,632	71,529
8‐10, n (%)	409 (1.1)	328 (1)	737 (1)
11‐13, n (%)	5635 (14.9)	4924 (14.6)	10,559 (14.8)
14‐15, n (%)	6096 (16.1)	5383 (16)	11,479 (16)
16‐17, n (%)	4474 (11.8)	3984 (11.8)	8458 (11.8)
18‐24, n (%)	8196 (21.6)	7408 (22)	15,604 (21.8)
25‐34, n (%)	5596 (14.8)	5040 (15)	10,636 (14.9)
35‐44, n (%)	3316 (8.8)	2984 (8.9)	6300 (8.8)
45‐54, n (%)	2061 (5.4)	1780 (5.3)	3841 (5.4)
55‐64, n (%)	1327 (3.5)	1121 (3.3)	2448 (3.4)
65+, n (%)	787 (2.1)	680 (2)	1467 (2.1)
Missing, n	593	572	1165
LGBTQ+^c^, n (%)	6524 (16.9)	5752 (16.8)	12,276 (16.9)
Student, n (%)	19,433 (50.5)	17,490 (51.1)	36,923 (50.8)
Trauma survivor, n (%)	5867 (15.2)	5363 (15.7)	11,230 (15.4)
Healthcare worker, n (%)	2117 (5.5)	1851 (5.4)	3968 (5.5)
New or expecting parent, n (%)	803 (2.1)	748 (2.2)	1551 (2.1)
Caregiver of someone living with emotional or physical illness^d^, n (%)	1415 (3.7)	1220 (3.6)	2635 (3.6)
Veteran or active-duty military, n (%)	997 (2.6)	835 (2.4)	1832 (2.5)
PHQ-9^e^, mean (SD)	15.8 (6.1)	15.9 (6.1)	15.9 (6.1)
PHQ-9 severity, n (%)			
Minimal (0‐4)	1494 (3.9)	1304 (3.8)	2798 (3.8)
Mild (5-9)	4969 (12.9)	4289 (12.5)	9258 (12.7)
Moderate (10-14)	9102 (23.6)	7948 (23.2)	17,050 (23.5)
Moderately severe (15-19)	11,176 (29)	10,090 (29.5)	21,266 (29.3)
Severe (20-27)	11,749 (30.5)	10,573 (30.9)	22,322 (30.7)

a Percentages for race and ethnicity, gender identity, household income, and age range are calculated out of available data; percentages for checkbox items (LGBTQ+, student, trauma survivor, healthcare worker, new or expecting parent, caregiver, and veteran) are calculated out of column totals.

b Original response options for gender identity included “female” and “male”; these are reported as “woman” and “man,” respectively.

c LGBTQ+: lesbian, gay, bisexual, transgender, and queer.

d The term “caregiver of someone living with emotional or physical illness” was not specifically defined; thus, participants may have interpreted this term in various ways (eg, healthcare professionals, adults caring for family members, daycare workers, and other paid or unpaid caregiving roles).

e PHQ-9: Patient Health Questionnaire-9

**Table 2.** Response proportions of intended next steps and perceived need items among participants who submitted the Next Steps survey.

Next Steps survey tailoring items and response options	Next Steps survey^a^ (n=34,204)
Intended next steps, n	33,002
Find a forum or support group for people with depression, n (%)	415 (1.3)
Find a treatment provider near you, n (%)	2334 (7.1)
I don’t want to do anything, n (%)	12,034 (36.5)
Learn about what therapy/treatment is like (reading articles), n (%)	1093 (3.3)
Learn and practice skills for how to manage depression (try a free self-help tool), n (%)	3463 (10.5)
Other, n (%)	1489 (4.5)
Take another mental health test, n (%)	4740 (14.4)
Tips for managing depression (reading articles), n (%)	3257 (9.9)
Understand and manage self-harm or suicidal thoughts, n (%)	1677 (5.1)
Understand what depression is like (reading articles), n (%)	2500 (7.6)
Missing, n	1202
Perceived need, n	33,352
No, n (%)	2453 (7.4)
I don’t know, n (%)	10,574 (31.7)
Yes, n (%)	20,325 (60.9)
Missing, n	852

a Percentages are calculated out of available data.

### Small Cost to Engagement by Adding Tailoring Questions

Table 3 displays logistic regression model estimates. Compared with the demographics survey condition, 625 more participants in the Next Steps survey condition disengaged from the postscreening survey web page. Disengagement rates were 25% for participants in the demographics survey condition and 27.5% for participants in the Next Steps survey condition. Participants who viewed the Next Steps survey had a 14% increase in the odds of disengaging from the postscreening survey (odds ratio [OR] 1.14, 95% CI 1.11‐1.16; *P*<.001) compared with participants who viewed the demographics survey.

**Table 3.** Logistic regression model estimates predicting the odds of disengaging (versus engaging) and clicking a featured resource (versus clicking a persistent general resource) by condition.

Predictors	Disengagement^a^		Click on featured resource^b^	
	OR^c^ (95% CI)	*P* value	OR (95% CI)	*P* value
Model 1: disengagement on the postscreening survey web page by Level 1 condition				
Demographics survey	Reference	Reference	—^d^	—
Next Steps survey	1.14 (1.11‐1.16)	<.001	—	—
Model 2: demographics survey; disengagement on the screening results page by Level 2 condition				
Nontailored	Reference	Reference	Reference	Reference
TR-D^e^	0.90 (0.87‐0.94)	<.001	1.90 (1.79‐2.01)	<.001
Model 3: Next Steps survey; disengagement on the screening results page by Level 2 condition				
Nontailored	Reference	Reference	Reference	Reference
TM-PN^f^	0.97 (0.91‐1.03)	.33	0.93 (0.85‐1.02)	.11
TR-INS^g^	1.02 (0.96‐1.08)	.51	1.25 (1.14‐1.37)	<.001
TM-PN+TR-INS^h^	0.98 (0.92‐1.04)	.52	1.34 (1.23‐1.47)	<.001
Model 4: optimal tailoring strategy; disengagement on the screening results page by Level 2 condition				
TR-D	Reference	Reference	Reference	Reference
Nontailored^i^	1.11 (1.07‐1.15)	<.001	0.52 (0.50‐0.55)	<.001
TM-PN	1.08 (1.02‐1.14)	.004	0.48 (0.45‐0.52)	<.001
TR-INS	1.13 (1.08‐1.19)	<.001	0.65 (0.60‐0.70)	<.001
TM-PN+TR-INS	1.09 (1.04‐1.15)	.001	0.70 (0.65‐0.75)	<.001

a Reference: engaged; Model 1: n=169,647; Model 2: n=38,490; Model 3: n=34,204; Model 4: n=72,694.

b Reference: click on persistent general resource; Model 2: n=20,939; Model 3: n=18,198; Model 4: n=39,137.

c OR: odds ratio.

d Not applicable.

e TR-D: tailored resources to demographics.

f TM-PN: tailored message to perceived need.

g TR-INS: tailored resources to intended next steps.

h TM-PN+TR-INS: tailored message to perceived need + tailored resources to intended next steps.

i Includes participants in both the demographics survey and Next Steps survey conditions (Level 1) who were subsequently randomized to the nontailored condition (Level 2).

### TR-D Reduced Disengagement and Increased Clicks on Featured Resources

Among participants who viewed the screening results page after submitting the demographics survey, those who received resources tailored to demographics had a 10% decrease in the odds of disengaging (OR 0.90, 95% CI 0.87‐0.94; *P*<.001) compared with participants in the nontailored condition. Participants in the TR-D condition had a 90% increase in the odds of clicking a featured resource versus a persistent general resource (OR 1.90, 95% CI 1.79‐2.01; *P*<.001) compared with participants in the nontailored condition.

### TR-INS and TM-PN+TR-INS Increased Clicks on Featured Resources

Among participants who viewed the screening results page after submitting the Next Steps survey, there were no significant differences in the odds of disengaging for participants in the TM-PN (*P*=.33), TR-INS (*P*=.51), and TM-PN+TR-INS (*P*=.52) conditions compared with the nontailored condition. However, there were statistically significant differences in the odds of clicking a featured resource versus a persistent general resource among those who engaged. Compared with the nontailored condition, those in the TR-INS condition had a 25% increase in the odds of clicking a featured resource (OR 1.25, 95% CI 1.14‐1.37; *P*<.001), and those in the TM-PN+TR-INS condition had a 34% increase in the odds of clicking a featured resource (OR 1.34, 95% CI 1.23‐1.47; *P*<.001). There was no significant difference in the odds of clicking a featured resource between the nontailored and TM-PN conditions (*P*=.11).

### Tailoring to Demographics Was the Optimal Tailoring Strategy in This Study

Tailoring resources to demographics yielded the best engagement outcomes compared with the remaining Level 2 conditions. Compared with participants shown resources tailored to demographics, those in the remaining conditions had 8%-13% greater odds of disengaging (*P*≤.004) and, among participants who engaged, had 30%-52% fewer odds of clicking a featured resource versus a persistent general resource on the screening results page (*P*<.001).

## Discussion

### Principal Findings

This study examined whether personalizing content increased engagement with a self-guided mental health website. There was a small but statistically significant cost to engagement from adding tailoring questions assessing perceived need and intended next steps, and tailoring resources to demographics was the most effective strategy used in this study. Appealing to perceived need alone via tailored messages, as chosen in this study based on HAPA theory, did not improve engagement. We speculate that this may be the case because although perceived need may be associated with intent to take action, intent is not necessarily associated with taking health actions (ie, the intention-behavior gap) [26]. Individuals with a perceived need to act may not be ready to do so immediately after viewing their screening results, and it is possible that increased cognitive load due to the additional text of tailored messages discouraged further engagement [27]. However, we also observed that while tailored resources to intended next steps increased the odds of clicking a featured resource by 25%, combining a tailored message to perceived need with tailored resources to intended next steps further increased those odds to 34%. It is possible that there was an interaction effect, such that tailored messages to perceived need may have facilitated engagement when individuals were also provided with resources relevant to their needs. Moreover, the HAPA has been used to describe behaviors that change over periods of days to weeks [18]. Whether the HAPA or other frameworks of health behavior change apply to long-term engagement with mental health websites remains unknown.

### Design Implications

Our findings have important generalizable design implications for implementing tailoring on websites to enhance engagement:

1. Defining engagement: we used indicators for disengagement and clicks on web-based resources to assess the impact of tailoring on early engagement. It is important that the operationalization of engagement aligns with the context of each study. For example, research questions related to long-term engagement may be better addressed by engagement outcomes such as total number of clicks, length of time spent on each web page, and whether visitors return to the website.
2. Selecting tailoring variables: tailoring variables may include, but are not limited to, any number of demographics, screening results, behavioral characteristics, and theoretical constructs of behavior change. Our preliminary MRTs identified variables that may be related to engagement specific to the MHA website. However, it is likely that engagement with websites that offer different types of resources or that are targeted toward specific populations is associated with variables not used for tailoring in this study. We recommend conducting pilot studies to identify potential tailoring variables related to engagement unique to each website. Researchers are also advised to consider the production costs of tailoring (eg, whether existing materials can be used or if new materials and design elements will need to be developed).
3. Acceptability of tailoring: researchers must also consider the acceptability of tailoring to demographics without suggesting that those characteristics are causal factors of visitors’ mental health problems [28]. For example, while tailoring to demographics was an effective strategy in this study, tailoring solely to LGBTQ+ status may have an inadvertent stigmatizing effect by implying to visitors that their depressive symptoms stem from their LGBTQ+ identity. Alternatively, researchers may ask participants about what they believe contributes to their mental health issues and tailor content accordingly.
4. Balancing costs and benefits: it is critical to identify the appropriate threshold for the number of tailoring questions to add to a website such that the costs of tailoring (ie, in this study, disengagement due to the addition of tailoring questions) do not outweigh the benefits. However, what constitutes costs and benefits depends on the goals of each website. For example, in some contexts, research questions may be better answered by retaining visitors who are willing to invest time and energy into answering many tailoring questions.
5. Randomization procedures: in the current study, participants were allocated to a Level 1 condition through simple randomization on the PHQ-9 web page, on which a link to the appropriate postscreening survey was generated. In addition, MHA does not retain screening data from visitors who do not submit a postscreening survey. Allocations to Level 1 conditions among participants who viewed a postscreening survey were slightly imbalanced (51.7% versus 48.3%) due to when randomization was triggered. To ensure balanced allocation to experimental conditions, future studies may use a 1:1 allocation among participants who meet inclusion criteria while taking existing data saving procedures into account.

### Strengths and Limitations

Our study was a large-scale randomized trial in an ecologically meaningful setting with a help-seeking population. Additional strengths include the use of objective measures of engagement and our design implications for implementing tailoring on websites. The generalizability of our findings may be limited to self-guided mental health websites, as opposed to other digital mental health formats (eg, apps and interventions).

One limitation of the study was that content could only be tailored for participants who responded to tailoring questions. By necessity, we could not assess the impact of tailoring for individuals who did not provide tailoring data. In addition, both featured resources and persistent general resources may have varied in appeal regardless of visitors’ personal characteristics and preferences. Nevertheless, our approach used MHA’s existing resources to align with the research questions as closely as possible in a real-world setting.

Another limitation was the use of IP addresses to identify individual website visitors. This method may not reliably distinguish between responses from multiple individuals accessing the website from within the same household or from visitors connected to a shared public network. However, MHA’s internal data cleaning procedures exclude entries from IP addresses of known public spaces (eg, libraries and schools; see Multimedia Appendix 1). Our analyses retained only the first entry from each unique IP address, which may have excluded data from additional visitors accessing the website from the same IP address. Nonetheless, our approach was a practical means of tracking website activity at a large scale, minimizing duplicate entries, and preemptively excluding responses from potential bots before analyses.

### Future Directions

This study focused on identifying strategies to enhance immediate engagement outcomes and was not designed to assess health behavior change over the long term. Tailoring to perceived need based on HAPA theory was effective in increasing the odds of clicking featured resources only when paired with tailored resources to participants’ intended next steps on the website. It is possible, however, that these decision processes are not well-suited for HAPA theory and that other guiding frameworks are needed for future research in this area. For example, other theoretical frameworks that have been used to tailor web-based interventions (eg, the theory of planned behavior [29], the transtheoretical model [30], and social cognitive theory [31]) may further guide research toward establishing optimal tailoring strategies on mental health websites. Future research may also test tailored messages that incorporate stronger calls to action intended to shift ambivalence (eg, via motivational interviewing language [32]). Given our evaluation of a restricted set of tailoring strategies, our findings provide an indication toward an optimal tailoring strategy for mental health websites. It is possible that other strategies not examined in this study, such as mode tailoring [33] or psychographic targeting [11], may also increase engagement in similar contexts. In addition, personal contexts and barriers can be assessed through open-ended questions to better understand contextual factors that may impact engagement with mental health websites, and long-term engagement patterns may be explored by identifying optimal times for re-engaging visitors when they are ready to access mental health resources.

### Conclusion

Mental health websites have the potential to serve millions of people with mental health concerns, particularly those who face barriers to treatment, yet their resources are underutilized. This study addresses the critical need for empirical research on effective strategies for connecting mental health website visitors with digital mental health resources. Our findings provide evidence that personalizing resources to demographic characteristics can enhance engagement with mental health websites: in this study, visitors who were shown resources tailored to demographics had 10% fewer odds of disengaging and, among those who engaged, 90% greater odds of clicking a featured versus a persistent general resource. We provide design implications for future research, which may explore alternative frameworks of behavior change, test different tailoring variables and strategies, and evaluate engagement over repeated visits. Researchers are encouraged to continue to address these gaps to improve engagement with mental health websites and better support individuals seeking web-based mental health information.

## Supplementary material

10.2196/73188 Multimedia Appendix 1 Mental Health America's data cleaning procedures.

10.2196/73188 Multimedia Appendix 2 Messages and featured resources.

10.2196/73188 Checklist 1 CONSORT-eHEALTH checklist (V 1.6.1)

## Abbreviations

HAPA: Health Action Process Approach
LGBTQ+: lesbian, gay, bisexual, transgender, and queer
MHA: Mental Health America
MRT: micro-randomized trial
OR: odds ratio
PHQ-9: Patient Health Questionnaire-9
SMART: sequential multiple assignment randomized trial
TM-PN: tailored message to perceived need
TM-PN+TR-INS: tailored message to perceived need + tailored resources to intended next steps
TR-D: tailored resources to demographics
TR-INS: tailored resources to intended next steps
