# Correction: Personalization Strategies for Increasing Engagement With Digital Mental Health Resources: Sequential Multiple Assignment Randomized Trial

**DOI:** 10.2196/88599

**Published:** 2025-12-11

**Authors:** Julien Rouvere, Isabell R Griffith Fillipo, Meghan Romanelli, Ashish Sharma, Brittany A Mosser, Theresa Nguyen, Kevin Rushton, John Marion, Tim Althoff, Michael D Pullmann

**Affiliations:** 1 Department of Psychiatry and Behavioral Sciences, University of Washington School of Medicine, 1959 NE Pacific Street, Box 356560, Seattle, WA, 98195-6560, United States, 1 206-221-5498; 2 School of Social Work, University of Washington, Seattle, WA, United States; 3 Paul G. Allen School of Computer Science and Engineering, University of Washington, Seattle, WA, United States; 4 Mental Health America, Alexandria, VA, United States

In “Personalization Strategies for Increasing Engagement With Digital Mental Health Resources: Sequential Multiple Assignment Randomized Trial” [1], the authors made one correction.

The Conflicts of Interest section has been changed from the following:

None declared.

The revised section now reads:

TN, KR, and JM are employees of Mental Health America. The remaining authors have no conflicts of interest to disclose.

The correction will appear in the online version of the paper on the JMIR Publications website, together with the publication of this correction notice. Because this was made after submission to PubMed, PubMed Central, and other full-text repositories, the corrected article has also been resubmitted to those repositories.
