# Supplementary material for: Personalization Strategies for Increasing Engagement With Digital Mental Health Resources: Sequential Multiple Assignment Randomized Trial
Source: JMIR Ment Health. 2025 Nov 4;12:e73188. doi: 10.2196/73188 (PMC12585131; doi:10.2196/73188)
Supplement: Multimedia Appendix 1 [file mental-v12-e73188-s001.docx]

## Multimedia Appendix 1

### Screening Data Download and Cleaning Procedure

We followed established best practices by Mental Health America (MHA) to meaningfully analyze the data. All activity on MHA is tied to visitors’ IP addresses. Data were cleaned by MHA using the following procedure prior to sharing the data with the University of Washington research team:

1. After raw monthly data were downloaded, IPs that were flagged as spam or bots in the past were excluded.
2. Duplicate entries and resubmissions were removed.
3. Entries identified as tests were removed.
4. IPs with >30 responses were checked to verify if they were spam and removed if so. IPs identified as spam were blocked so that data will not be collected from them in the future.
5. Identifiable information was removed (ie, names, phone numbers, addresses, email addresses).
6. Only domestic data were retained (ie, data from IPs that answered “Do you live in the United States or another country?” with “I live in the United States” or “My child lives in the United States”). Data from IPs that selected “I live in another country,” “My child lives in another country,” or did not provide data on location were removed.
7. MHA only saves screening data (eg, Patient Health Questionnaire-9 results) from visitors who submit a postscreening survey.

### Bot Cleaning Process

IPs that were flagged as likely bots were identified via server logs. MHA identified a group of tags (eg, SiteImprove, SemanticScholarBot, or GoogleBot) that identify bots, webscrapers, or other non-human traffic by searching through MHA’s logs and looking for strange behavior patterns. IPs flagged as bots are saved, and screens from those IPs are removed, which amount to roughly 2-3% of screens. MHA is increasingly on the watch for bot activity and uses the safest, most conservative approach for identifying and removing data from bots.
