# Supplementary material for: Personalization Strategies for Increasing Engagement With Digital Mental Health Resources: Sequential Multiple Assignment Randomized Trial
Source: JMIR Ment Health. 2025 Nov 4;12:e73188. doi: 10.2196/73188 (PMC12585131; doi:10.2196/73188)
Supplement: Multimedia Appendix 2 [file mental-v12-e73188-s002.docx]

## Multimedia Appendix 2

### Messages and Featured Resources

Messages and featured resources shown on the screening results page varied by condition and by the types of tailoring information provided by participants. Participants who did not provide tailoring information were shown nontailored messages or resources, depending on their Level 2 condition. For example, participants in the tailored message to perceived need (TM-PN) condition who did not provide a response to the perceived need tailoring item were shown a nontailored message and nontailored featured resources.

See the following tables for messages and featured resources used in this study:

- Table 1: Nontailored messages (shown across all conditions).
- Table 2: Tailored messages to perceived need: “Do you feel like you need to do something to improve your mental health?”
- Table 3: Nontailored featured resources.
- Table 4: Tailored resources to demographics (age and LGBTQ+ status).
- Table 5: Tailored resources to intended next steps: “What is the main thing you want to do after taking this mental health test?”

Table 1. Nontailored messages (shown across all conditions).

| **Depression severity** | **Nontailored message (ie, brief description of PHQ-9^a^ results)** |
| --- | --- |
|  |  |
| Minimal depression  (PHQ-9=0-4) | Based on your responses, you have few or no symptoms of depression. This result is not a diagnosis. A doctor or therapist can help you get a diagnosis and/or treatment. |
| Mild depression  (PHQ-9=5-9) | Based on your responses, you may have symptoms of mild depression. This result is not a diagnosis. A doctor or therapist can help you get a diagnosis and/or treatment. |
| Moderate depression  (PHQ-9=10-14) | Based on your responses, you may have symptoms of moderate depression. This result is not a diagnosis. A doctor or therapist can help you get a diagnosis and/or treatment. |
| Moderately severe depression  (PHQ-9=15-19) | Based on your responses, you may have symptoms of moderately severe depression. This result is not a diagnosis. A doctor or therapist can help you get a diagnosis and/or treatment. |
| Severe depression  (PHQ-9=20-27) | Based on your responses, you may have symptoms of severe depression. This result is not a diagnosis. A doctor or therapist can help you get a diagnosis and/or treatment. |

^a^PHQ-9: Patient Health Questionnaire-9.

Table 2. Tailored messages to perceived need: “Do you feel like you need to do something to improve your mental health?”

| **Response** | **Tailored message to perceived need** |
| --- | --- |
|  |  |
| **Beginning text based on response to perceived need** | |
| Perceived need=“No” or “I don’t know” | It sounds like you’re not sure if you need to do something to help you feel better. Not everyone feels ready to seek mental health treatment right away. |
| Perceived need=“Yes” | We're so glad to hear you're open to exploring how to improve your mental health. |
| **Following text based on depression severity** | |
| Minimal or mild depression | People who score with minimum or mild depression often notice that symptoms/experiences can get worse in the weeks after taking a Depression Test.  Check out the resources below or take another mental health test (like for anxiety). They've helped people facing similar challenges. |
| Moderate, moderately severe, or severe depression | Many people who score with moderate to severe depression notice that suicidal thoughts and difficulty with managing life can get worse in the weeks after taking a Depression Test.  Check out the resources below based on your responses. They've helped other people facing similar challenges. |

Table 3. Nontailored featured resources.

| **Condition** | **Nontailored featured resources** |
| --- | --- |
|  |  |
| Always shown to participants in the following conditions:   - Nontailored - TM-PN^a^   Conditionally shown to participants in:   - The TR-D^b^ condition who did not provide LGBTQ+^c^ status or did not self-identify as LGBTQ+ and did not provide age range - The TR-INS^d^ and TM-PN+TR-INS^e^ conditions who did not provide a response to the intended next steps item or who responded “Other” or “I don’t want to do anything” | - Overcoming negative thoughts - Does depression go away on its own? - “I can’t get out of bed” or “How to treat depression” - “Are there types of depression” or “Am I depressed or just sad?” |

^a^TM-PN: tailored message to perceived need.

^b^TR-D: tailored resources to demographics.

^c^LGBTQ+: lesbian, gay, bisexual, transgender, and queer.

^d^TR-INS: tailored resources to intended next steps.

^e^TM-PN+TR-INS: tailored message to perceived need + tailored resources to intended next steps.

Table 4. Tailored resources to demographics (age and LGBTQ+ status).

| **Demographic characteristic** | **Tailored resources pool** |
| --- | --- |
|  |  |
| **Age range (years)** | |
| Youth (8-17) | - I think about death all the time - What mental illness do I have? - I hate myself - How can I get help without my family knowing? |
| Young adults (18-24) | - I don’t want to live, but I don’t want to die - Am I depressed or just sad? - Feeling lonely in a crowd - I don’t want to leave my room |
| Middle adults (25-45) | - I can’t do anything right—depression - Does depression go away on its own? - I feel numb - I can't get out of bed |
| Adults (45+) | - Are there types of depression? - How can I improve my mental health on my own? - Thinking traps: How can I deal with negative thoughts? |
| **LGBTQ+^a^ status** | |
| LGBTQ+ (youth or blank age) | - I’m bullied because I’m LGBTQ+/queer - Q-chat space - Prepare for difficult conversations - How do I find LGBTQ-friendly therapy? |
| LGBTQ+ (18+) | - I’m bullied because I’m LGBTQ+/queer - Prepare for difficult conversations - How do I find LGBTQ-friendly therapy? |

^a^LGBTQ+: lesbian, gay, bisexual, transgender, and queer.

Table 5. Tailored resources to intended next steps: “What is the main thing you want to do after taking this mental health test?”

| **Response** | **Tailored resources** |
| --- | --- |
|  |  |
| Take another mental health test | - Take a Mental Health Test - What mental illness do I have? - How do I know if I have a mental illness? - I just took a mental health test. What next? |
| Understand what depression is like (reading articles) | - What is depression really like? - Am I depressed or just sad? - Are there types of depression? - Am I broken? |
| Understand and manage self-harm or suicidal thoughts | - I don’t want to live, but I don’t want to die - I think about death all the time - How can I tell someone I’m harming myself? - How do I stop harming myself? |
| Tips for managing depression (reading articles) | - How to get out of a funk - Thinking traps: How can I deal with negative thoughts? - I can’t stop snapping at people - I can’t get over things that happened in the past |
| Learn and practice skills for how to manage depression (try a free self-help tool) | - Getting Unstuck: Practice Changing Negative Thoughts - Overcoming negative thoughts - Addiction: What’s keeping me stuck - DIY Tools |
| Learn about what therapy/treatment is like (reading articles) | - Who can I talk to about my mental health? - How do you treat depression? - Does depression go away on its own? - How does therapy work? What to expect |
| Find a treatment provider near you | - Get Help - Need to talk to someone? (Warmlines) - FindTreatment.gov - How do I find a therapist? |
| Find a forum or support group for people with depression | - How can connecting with others help my mental health? - Mental Health Support Groups - ADAA Online Peer-to-Peer Anxiety and Depression Support Group - Mental health forum: MHA on Inspire |
